# The association between childhood conditions and heart disease among middle-aged and older population in China: a life course perspective

**DOI:** 10.1186/s12877-021-02134-9

**Published:** 2021-03-17

**Authors:** Jingyue Zhang, Nan Lu

**Affiliations:** 1 Institute of Gender and Culture, Changchun Normal University, Changji North Road 677, Changchun, 130052 Jilin Province China; 2 Department of Sociology, School of Philosophy and Sociology, Jilin University, Qianjin Street 2699, Changchun, 130012 Jilin Province China; 3 Department of Social Work and Social Policy, School of Sociology and Population Studies, Renmin University of China, No. 59 Zhongguancun Street, Haidian District, Beijing, 100872 China

**Keywords:** Childhood conditions, Heart disease, Life course, CHARLS

## Abstract

**Background:**

Heart disease is a severe health problem among adult populations in China. The prevalence rates of heart disease increase with age. The pathogenic causes of heart disease are often related to conditions in early life. Using a nationally representative sample of adults aged 45 or older in China, we examined the association between childhood conditions and heart disease in later life from a life course perspective.

**Methods:**

The data used in this study were derived from the life history module and 2015 wave of China Health and Retirement Longitudinal Study (CHARLS). Missingness were handled by multiple imputation, generating 20 complete datasets with a final sample of 19,800. Doctor-diagnosed heart disease was the main dependent variable. Respondents’ conditions in childhood, adulthood, and older age were the independent variables (e.g., socioeconomic status, health, and health resources). Random-effects logistic regression models were conducted to test the hypotheses.

**Results:**

A total of 16.6% respondents reported being diagnosed with heart disease by doctors. Regarding childhood socioeconomic status, 8.2% of the respondents considered that they were (a lot) better off than their neighbors, and 31.1% considered that their health status in childhood was better than their peers. More than 90% of respondents did not have severe illnesses during their childhood, and around 80.3% had access to health resources nearby in childhood. Lower socioeconomic status and poorer health conditions in childhood were associated with a greater likelihood of reporting doctor-diagnosed heart diseases, even after controlling for conditions in adulthood and older age (socioeconomic status: odds ratio (OR) = 0.947; self-rated health: OR = 0.917; severe illnesses: OR = 1.196).

**Conclusions:**

Along with chronic diseases (e.g., hypertension, diabetes, and dyslipidemia), unhealthy behaviors, overweight and obesity, poor childhood conditions should be considered as screening criteria to identify populations at risk of heart disease. Relevant preventive strategies and interventions should be developed from a life course perspective and conducted in communities by providing health education program among older population with low socioeconomic status, and encouraging early detection and treatment.

## Background

Heart disease refers to a set of heart problems, including but not limited to coronary heart disease, heart attack, congestive heart failure, and angina [1]. Heart disease accounted for around 40% of disease-related deaths in the Chinese population [2]. It is not only the top cause of mortality, which is even higher than cancer and cerebrovascular diseases [3]. Heart disease not only has adverse impacts on individuals’ physical health and social functioning, but also is a risk factor for Alzheimer disease and other types of dementia [4, 5]. The prevalence of heart disease has been increased steadily in China (e.g., around 15% increase in age-standardized prevalence rate from1990 to 2016), and is predicted to continue to rise in the next few decades [3]. At the same time, hospitalization costs due to heart disease are increasing rapidly [2]. Under such circumstances, China has encountered great social and economic burdens due to heart disease. There is an urgent need for preventive strategies and interventions that promote heart health and healthy aging among community-dwelling older adults.

Previous research identified a range of individual-level social and health factors related to heart disease [6–10]. Specifically, recent Chinese studies identified significant associations between unhealthy behaviors (e.g., smoking) and heart disease [6, 7, 9]. Overweight and obesity were found to be key risk factors of hypertension and dyslipidemia, which further increase the risk of heart disease [1, 11]. Physical inactivity was found to be another risk factor of heart disease [1, 12].

Childhood conditions could also be important determinants of heart diseases in later life. Literature has shown that childhood conditions are associated with a range of health outcomes in later life, including functional health, mental health, cognitive capacities, chronic diseases, and even mortality [13–15]. Some studies, for example, suggested that early life conditions play an important role in influencing functional health status in later life in developing countries [13]. Another Chinese study found that early life conditions affected cognitive function in middle-aged and older adults [15]. Furthermore, children from poor families were more likely to have low educational attainment, low income, and poor health status in later life than their counterparts from rich families [16]. Therefore, we argue that risk factors and behaviors related to heart disease could be traced back to childhood, and the effects might accumulate over time and influence adulthood and even older life [17–19].

There are an increasing number of studies examining different sets of risk factors and behaviors linked to heart disease at different life course stages [18, 20]. However, only limited number of studies explored the relationships between childhood conditions and heart disease in the context of China. Among these studies, the majority focused on the effects of SES on heart disease in later life. Few studies have considered other childhood conditions such as health conditions and access to health resources [19, 21–23]. Using multiple childhood indicators could not only provide more comprehensive measurements, but also allow us to test whether different childhood conditions could have different influences on heart disease in later life. Therefore, the present study focused on multiple childhood conditions, including childhood SES, childhood self-rated health (SRH), severe illness in childhood, and access to health resources nearby in childhood [16, 19]. Given the trend toward younger people being diagnosed with heart disease in China, this study aimed to examine early life risk factors of heart disease among middle-aged and older populations.

### Early-life conditions and heart disease in later life

The pathways linking childhood conditions to heart disease in later life can be both direct and indirect. Two main theoretical perspectives were used to explain the association between childhood conditions and health outcomes in later life: the latency model and the pathway model [24–27]. The latency model suggests that childhood circumstances have a direct impact on health outcomes in adulthood and later life through influencing individuals’ health trajectory patterns [15, 28]. In other words, childhood adversities such as low SES, undernutrition, and severe illness may permanently alter individuals’ trajectories of health throughout the life course, even after controlling for conditions in adulthood. For instance, poor maternal nutrition in childhood could weaken individuals’ immune systems and alter the function of important organs associated with blood pressure, lipid regulation, and insulin [16]. In turn, this could increase the risk of chronic diseases in adulthood and later life, such as heart disease and diabetes [26, 29, 30].

Furthermore, the pathway model argues that childhood circumstances could indirectly affect health outcomes in later life. Childhood adversity often has adverse impacts on health outcomes in later life through influencing individuals’ educational achievement and employment in adulthood [14, 30]. Poor childhood conditions could increase the risk of chronic diseases in adulthood (e.g., hypertension and heart disease), which in turn could have long-term adverse impacts on their cognitive function and social competence and further lead to relatively low levels of educational achievement, occupational status, and income in adulthood [14, 19, 30]. In addition, low SES in childhood and adulthood might be associated with poor living environment and living conditions, which can further affect individuals’ educational achievement and health outcomes in later life [26, 30]. Childhood conditions are also associated with health-related behaviors. Individuals raised in families with low SES are more likely to adopt unhealthy behaviors, such as smoking and alcohol use, which can affect health outcomes in later life [13, 31]. It is important to note that the latency model and the pathway model are not mutually exclusive. Both processes could coexist and influence health outcomes in later life.

The literature has a few major gaps concerning childhood conditions and heart disease and other health outcomes in later life. First, many previous studies used selective samples (e.g., oldest-old adults and samples from hospitals) [5, 20, 32]. Therefore, the empirical generalization of the findings are limited. Second, although different childhood conditions might affect health in later life differently, the majority of studies only used childhood SES to represent childhood conditions [15, 22, 25, 27]. Third, most previous studies were conducted in developed countries; relatively less is known about whether the association between childhood conditions and heart disease exists in developing countries [22, 25]. It is important to note that the childhood conditions of a large proportion of current middle-aged and older cohorts are harsher than their counterparts in developed countries [13]. Therefore, new empirical evidence is needed to inform policy and intervention developments in developing countries, especially in China, which has the largest middle-aged and older population in the world.

China has undergone dramatic transitions, both socially and economically, mainly due to economic reforms and rapid urbanization in the past few decades. Before the economic reforms implemented in the late 1970s, China had undergone the Great Chinese Famine (1959–1961) and the Great Cultural Revolution (1966–1976). Thus, many middle-aged and older adults born before 1970 experienced unrest and upheaval during childhood. After the economic reforms, millions of Chinese adults’ standards of living have improved significantly [33]. Although improved living standards are important in influencing health outcomes in later life, this may not completely offset the disadvantages of childhood conditions on health outcomes in later life.

In the present study, we used the China Health and Retirement Longitudinal Study (CHARLS) to examine whether childhood conditions are associated with the onset of heart diseases in later life among middle-aged (45–59 years) and older (60 years or older) adults in China. CHARLS is a nationally representative survey of the population aged 45 or older. A life history survey was conducted in 2014, so CHARLS is suitable to examine the association between childhood conditions and heart diseases through a life course perspective. We extended previous literature by investigating the effects of childhood SES, childhood SRH, illnesses in childhood, and access to health care resources in childhood on heart disease in later life. Furthermore, we tested the roles of illness status, access to health care resources, body mass index (BMI), functional loss, and hypertension in adulthood as conditions in later life. At the same time, we controlled for demographic characteristics and health-related behaviors. It is important to note that China is a two-tier society. This means that in China, citizens living in urban and rural areas hold different household registration types: nonagricultural and agricultural, respectively. Compared to citizens with an agricultural household registration, those with a nonagricultural household registration status usually have more educational opportunities and access to better medical resources and social welfare systems [34–36]. Therefore, we added household registration status as a control variable.

In summary, poor living conditions, low SES, and poor health in childhood could have direct and indirect negative effects on health outcomes in later life [22, 25, 27]. Findings regarding the association between childhood conditions and heart disease in later life are important for policy and intervention implications and could further help China and other developing countries achieve healthy and active aging. Based on the literature and theoretical models previously discussed, we proposed the following hypotheses about the relationship between childhood conditions and heart disease in later life among Chinese older adults:

1. Low socioeconomic status in childhood is associated with more heart disease in later life.
2. Poor childhood self-rated health is associated with more heart disease in later life.
3. Severe illnesses in childhood are associated with more heart disease in later life.
4. Low levels of access to health care resources in childhood are associated with more heart disease in later life.

## Methods

### Sampling

The data of the study were derived from the CHARLS, a national longitudinal survey of Chinese community-dwelling adults aged 45 or older; it is a publicly available dataset [37]. CHARLS offers a wide range of information on individuals aged 45 or older and their household demographics, SES, family structure, and health circumstances. The baseline of CHARLS was performed in 2011, using multistage stratified probability-proportionate-to-size sampling to recruit about 10,000 households in 28 provinces. Face-to-face computer-assisted interviews were used to collect information about the respondents, and the respondents were tracked every two to three years.

For this analysis, 2014 life history data and the 2015 wave of the survey were used. The 2014 life history data were collected in a special module aimed to study the life course of the respondents. The life history survey questionnaire of 2014 included abundant information about the individual’s childhood family conditions, education, pregnancy, health, health care, wealth, and work history; 20,543 respondents who had completed the 2011 and 2013 waves completed the questionnaire of life history. In this analysis, the basic information of the respondents was derived from 2015 wave, and information about their childhood and adulthood was derived from the 2014 life history module. The number of eligible respondents aged 45 years and older from 2015 wave was 19,800. The missingness were handled by using multiple imputation to create 20 complete datasets of the 19,800 respondents (please see the data analysis section below).

### Measurement

#### Dependent variable

In this analysis, doctor-diagnosed heart disease was used as the outcome variable. Respondents indicated whether they had been diagnosed with heart disease (i.e., heart attack, coronary heart disease, angina, congestive heart failure, or other heart problems) by a doctor? The answer options were “yes” or “no,” creating a binary variable (1 = *yes*, 0 = *no*).

#### Childhood conditions

Four childhood conditions were added to the model: childhood SES, childhood SRH, illnesses in childhood, and health care resources in childhood. In the life history survey of CHARLS, a question measured respondents’ childhood SES: “Compared to the average family in the same community/village before age 17, how was your family’s financial situation?” The answers were measured on a 5-point Likert scale: 1 = *a lot better off than them*; 3 = *same as them*; 5 = *a lot worse off than them*. For convenience, we reversed the scale and recoded the values as 0–4; greater values indicated better childhood SES. Childhood SRH was measured by a single question: “Before or when you were 15 years old, compared to your peers, how was your health status?” The answers were measured on a 5-point Likert scale: 1 = *much healthier*; 3 = *about average*; 5 = *much less healthy*. We also reversed the scale as 0–4. Greater values indicated better childhood SRH. Illnesses in childhood were measured by asking respondents whether because of health conditions they ever missed school, were confined to bed or home, or were hospitalized for one month or longer before they turned 16. The answer of “yes” was coded as 1, and the answer of “no” was coded as 0. We added the values of the three conditions to create a sum score ranging from 0 to 3, and recoded the variable as a binary variable (0 = *0*, 1 = *1–3*). Health care resources in childhood were measured by asking respondents when they were 15 or younger and were sick or needed health advice, whether they had a usual source of care (such as a particular person or place) within 2 h of their home. The answer of “yes” was coded as 1, and the answer of “no” was coded as 0.

#### Adulthood conditions

Illnesses in adulthood were measured by asking respondents whether because of health conditions they had ever been confined to bed or home, hospitalized, left their job for one month or longer after they turned 16. We added the values of the three conditions, creating a sum score ranging from 0 to 3, and further recoded the variable as a binary variable (0 = *0*, 1 = *1–3*). Health care resources in adulthood were measured by asking respondents whether they had a usual source of care after they turned 16 and were sick or needed health advice. The answer of “yes” was coded as 1, and the answer of “no” was coded as 0.

#### Conditions in later life

In the third model, we added respondents’ household assets, BMI, functional loss, hypertension status, and health care resources as conditions in later life. Household assets included respondents’ property valuation, mortgage for their primary house, fixed capital assets, vehicles, livestock, other real estate, nonfinancial household assets, and arable land. We used a general formula to calculate BMI: weight divided by height squared. Then we recoded the value of BMI as an ordinal variable according the general standard: BMI < 18.5 was defined as underweight; 18.5 ≤ BMI < 25 was defined as normal weight; 25 ≤ BMI < 30 was defined as overweight; and BMI > 30 was defined as obesity. The BMI value range of normal weight was the reference group. Functional loss was defined as having difficulty with walking 100 m; walking or running about 1 km; climbing several stairs without resting; kneeling, stooping, or crouching; reaching or extending arms above shoulder level; and after sitting for a long period, getting up from a chair. The answer of “yes” was coded as 1, and the answer of “no” was coded as 0. We summed the scores for all questions related functional loss to represent the respondents’ functional status; with greater scores indicating worse functional status. The staus of hypertension, diabetes, dyslipidemia were measured by asking respondents whether they had ever been diagnosed with the above diseases by a doctor. Also, health care resources in later life were measured by asking respondents whether they had a usual source of care when they were sick or needed health advice. For these questions, the answer of “yes” was coded as 1, and the answer of “no” was coded as 0.

#### Control variables

We added age, sex, education level, marital status, household registration status, and health-related behaviors into the model as control variables. Age was measured by subtracting the year of birth of the respondents from the survey date. Sex, marital status, and household registration status were coded as binary variables. For sex, female was coded as 0 and male was coded as 1. For marital status, married was coded as 1, and other marital status was coded as 0. For household registration status, agricultural household registration was coded as 0, and nonagricultural household registration was coded as 1. Education level was measured by asking respondents to report the highest level of education they had attained. We recoded education level as a binary variable: 0 represented no formal education (illiterate), and 1 represented primary school or home school education or beyond. Furthermore, respondents were asked whether they ever smoked or consumed any alcoholic beverages. For these two questions, 0 represented no use, whereas 1 represented any use.

### Data analysis

In this analysis, we handled missing data through the method of multiple imputation by chained equations (MICE) in Stata 15.0 statistical software. This method allows researchers to conduct separate conditional distributions for different types of imputed variables. Therefore, it is suitable to conduct imputations on binary outcome variables in longistic regression models [38, 39]. In this study, all variables except age, sex, and marital status had missing data. The percentages of missingness ranged from 0.1 to 22.8%. We selected auxiliary variables, including activities of daily living, body pain, cognitive function, and depressive symptoms. These variables were not included in the final analysis. They were related to the missing variables and provided additional information to conduct multiple imputation. All variables with missing data were imputed. The final sample size of the 2015 wave in CHARLS is 19,800. A total of 20 complete datasets were created to further conduct statistical analysis.

The basic unit of CHARLS is a household. In the 2015 wave, about 10,000 households were selected. Therefore, we used random-effects logistic regression models to test the effects of childhood, adulthood, and later-life conditions on heart disease among older adults. We conducted three models to test the effects. In the first model, we entered childhood condition and control variables into the model. In the second model, we added adulthood condition variables to test the effect of adulthood conditions on the outcome variable after controlling childhood condition and other control variables. In the third model, we added variables of late-life conditions into the model. The models were conducted in Stata 15.0.

## Results

### Descriptive statistics

The descriptive statistical results of variables are shown in Table 1. We used frequency to describe categorical variables and mean to describe continuous variables. In this sample, 16.6% of respondents were diagnosed with heart disease by a doctor. The mean age was 60.2 years. More than 50% respondents were female. The percentage of married respondents was 86.4%. More than 70% of respondents had received formal education, and 72.8% held an agricultural household registration. Furthermore, the mean scores of SES and SRH in childhood were 1.47 and 2.34, respectively. During childhood, more than 90% of respondents did not miss school or were not hospitalized or confined to bed for a month or longer. This figure decreased to around 80% in adulthood. The percentage of health care resources increased over time, from 80.3% in childhood to 81.8% in adulthood and 87.3% in later life. The mean scores of BMI and functional loss were 23.9 and 0.42, respectively. Finally, 31, 16.1, and 9.3% of the respondents had hypertension, diabetes, and dyslipidemia, respectively.

**Table 1:** Sample Characteristics (*N* = 19,800)

	N (%)	Mean (SD)	Missing rate (%)
**Had Heart diseases**	3293 (16.6)		15.2%
**Childhood conditions**			
SES		1.47 (0.98)	11.9%
SRH		2.34 (1.01)	12.0%
Illnesses	1508 (7.6)		11.8%
Had resources of health care	15,905 (80.3)		11.1%
**Adulthood conditions**			
Illnesses	4003 (20.2)		11.4%
Had resources of health care	16,197 (81.8)		11.1%
**Later life conditions**			
Household assets		5365.9 (16,548.1)	3.6%
BMI		23.9 (3.9)	22.8%
Functional loss		0.42 (1.06)	3.2%
Had hypertension	6129 (31.0)		14.5%
Had dyslipidemia	3186 (16.1)		17.1%
Had diabetes	1841 (9.3)		15.8%
Had resources of health care	17,278 (87.3)		11.1%
**Covariates**			
Age		60.2 (10.2)	0.0%
Sex			0.0%
Female	10,161 (51.3)		
Male	9639 (48.7)		
Marital status			0.0%
Married	17,105 (86.4)		
Other marital status	2694 (13.6)		
Education			0.1%
Illiterate	5010 (25.3)		
Had received formal education or higher	14,762 (74.6)		
Household registration			7.5%
Agriculture	14,412 (72.8)		
Non-agriculture	3902 (19.7)		

Note: *SES* Socioeconomic Status, *SRH* Self-rated health

Illnesses = Being confined to bed or home/hospitalized/missed school/left job for more than one month

### Regression models

As discussed, we used random-effects logistic regression to test the effect of childhood conditions on heart disease, and the results are shown in Table 2. In Model 1, we entered childhood variables and control variables. The results show that all conditions in childhood influenced heart disease significantly. Respondents with good SES and SRH during childhood were less likely to have heart disease (SES: OR = 0.935; SRH: OR = 0.936). Respondents who had more health care resources during childhood also had lower risk of heart disease (OR = 0.770). However, respondents who ever missed school or were hospitalized or confined to bed or home for a month or longer during childhood were more likely to have heart disease (OR = 1.341).

**Table 2:** Random-effects Logistic Regression for Life Course Conditions and Heart Disease

	Model 1			Model 2			Model 3		
	***B***	***SE***	OR	***B***	***SE***	OR	***B***	***SE***	OR
**Childhood conditions**									
SES	−0.067	0.024	0.935*	− 0.055	0.024	0.946*	− 0.054	0.025	0.947*
SRH	−0.066	0.023	0.936*	− 0.051	0.023	0.951*	−0.087	0.024	0.917*
Illnesses	0.293	0.079	1.341*	0.198	0.080	1.219*	0.179	0.082	1.196*
Resources of health care	−0.262	0.075	0.770*	−0.118	0.143	0.889	−0.123	0.138	0.884
**Adulthood conditions**									
Illnesses				0.622	0.052	1.862*	0.472	0.055	1.603*
Resources of health care				−0.157	0.156	0.854	−0.226	0.158	0.798
**Later life conditions**									
Household Asset							−0.011	0.011	0.989
BMI (Normal weight)									
Underweight							0.073	0.106	1.075
Overweight							0.220	0.057	1.247*
Obesity							0.493	0.098	1.638*
Functional loss							0.090	0.020	1.095*
Hypertension							0.893	0.051	2.443*
Dyslipidemia							0.857	0.056	2.356*
Diabetes							0.386	0.067	1.471*
Resources of health care							0.348	0.175	1.416*
**Covariates**									
Age	0.057	0.003	1.058*	0.055	0.003	1.056*	0.043	0.003	1.044*
Age square	−0.002	0.000	0.998*	−0.002	0.000	0.998*	−0.001	0.000	0.999*
Sex	−0.803	0.070	0.448*	−0.823	0.071	0.439*	−0.762	0.073	0.467*
Marital status	−0.047	0.066	0.954	−0.044	0.066	0.957	−0.033	0.067	0.967
Education	0.255	0.056	1.290*	0.265	0.056	1.303*	0.236	0.058	1.266*
Household registration	0.618	0.056	1.856*	0.610	0.057	1.840*	0.446	0.059	1.562*
Smoking	0.323	0.065	1.381*	0.318	0.065	1.374*	0.373	0.068	1.451*
Drinking	−0.056	0.052	0.945	−0.054	0.052	0.948	−0.071	0.053	0.931

Note: *SES* Socioeconomic Status, *SRH* Self-rated health

Illnesses = Being confined to bed or home/hospitalized/missed school/left job for more than one month

* = *p* < .05

In Model 2, we added adulthood variables. Except health care resources, all other childhood variables remained significant. For adulthood variables, health care resources were also nonsignificant. Respondents who had ever been confined to bed or home, were hospitalized, or left their job for a month or longer during adulthood were more likely to have heart disease (OR = 1.862).

We further entered variables of later-life conditions in Model 3. SES, SRH, childhood illnesses, and adulthood illnesses were significantly associated with heart disease. Regarding variables of later-life conditions, as compared with respondents with normal weight, respondents with higher BMI values were more likely to have heart disease (overweight: OR = 1.247; obesity: OR = 1.638). Respondents with worse functional status and higher hypertension, diabetes, and dyslipidemia had more risk of heart disease (functional loss: OR = 1.095; hypertension: OR = 2.443; diabetes: OR = 1.471; dyslipidemia: OR = 2.356). Finally, respondents who had higher access to health care resources in later life were less likely to have heart diseases (OR = 1.416).

## Discussion

The findings highlight the pivotal role of childhood SES, childhood health status, and health care resources in risk of heart disease among Chinese populations. Some findings of this study are consistent with prior studies [6, 9, 12, 18, 19, 22]; age was the most important predictor of morbidity related to heart disease. However, sex, education level, household registration status, overweight, obesity, hypertension, unhealthy behaviors (e.g., smoking), and functional loss in later life were also significantly associated with heart disease morbidity in later life. Considering childhood conditions, consistent with prior studies [22, 25, 27], in this study, childhood SES was a significant predictor of heart disease. However, this study extended the empirical evidence, suggesting that childhood SRH, severe illnesses in childhood, access to health care resources during childhood, and severe illnesses in adulthood also have significant effects on heart disease morbidity in later life, after controlling childhood SES in the model.

Additionally, childhood SES, childhood SRH, and severe illnesses in childhood remained significant determinants of heart disease in later life after we added adulthood conditions and variables in later life to the model. The association between access to health care resources during childhood and heart disease in later life, however, became statistically nonsignificant when we added adulthood conditions to the model. That means this relationship could be explained through severe illnesses in adulthood. The findings support both the latency model and the pathway model; childhood SES, childhood SRH, and severe illnesses in childhood had direct impacts on heart disease in late life, whereas access to health care resources during childhood had indirect impacts on heart disease in late life.

The findings of this study have policy and intervention implications. First, adults with poor childhood SES, poor childhood and health status, no health care resources during childhood, overweight or obesity, hypertension, diabetes, dyslipidemia, and unhealthy behaviors should be screened to confirm whether they have potential risk of heart disease. Given the high morbidity of heart disease and its great social and economic costs, policy makers should develop policies that support heart disease prevention and health education programs (e.g., choosing a healthy diet, exercising regularly, and maintaining a healthy weight) among high-risk populations, especially those with lower educational achievement. At the same time, policy makers should reduce the hospitalization costs of heart disease and encourage early detection and treatment. Second, because the development of heart disease usually begins in early life and risk factors for heart disease are identifiable in childhood [18], policy makers and intervention designers should use a life course perspective to improve prevention strategies related to heart disease and start interventions in childhood. Reasonable strategies for preventing heart disease beginning in childhood could detect risk factors and then prevent them from progressing [17]. And for individuals who are confirmed to have diseases that could predispose them to heart disease, diagnosis in early life could ensure they receive early treatment, which could reduce the risk of heart disease in later life [18].

Finally, evidence increasingly suggests that the prevention strategies for populations at risk of heart disease should focus on childhood conditions as well as some key factors in adulthood and later life (e.g., adulthood health status and access to health care resources nearby in later life) [11, 18]. Detecting them in an earlier life stage could help individuals delay the onset of heart diseases in later life. Thus, policy makers should help individuals maintain lifelong ideal heart health through developing prevention programs for health management and changing educational approaches and the environment to prevent chronic diseases.

Although the present study has many merits, it is not without limitations. First, the life history data depended on a retrospective survey. More accurate and objective data should be collected in future longitudinal studies. Second, biological inheritance is a risk factor for heart disease; however, information on family health history and genetic factors were not included in the CHARLS questionnaires. Thus, we could not control for biological inheritance in the analysis. Third, the retrospective results of SRH status in the data may lead to inaccuracy and misclassification bias. Fourth, future longitudinal studies should use valid scales to provide more accurate and comprehensive measurements of conditions in childhood, adulthood, and later life, as well as the status of chronic diseases. Fifth, medical care resources in rural areas are limited, and some rural residents with heart disease may not have been diagnosed. Therefore, the morbidity of heart disease among rural respondents might be underestimated. In this case, the relationship between childhood conditions and heart diseases might be underestimated. Sixth, this study mainly focused on the relationship between childhood conditions and heart diseases. Furture studies should further explore the potential role of race, the history of drinking and smoking habits (e.g., being heavy drinker and smoker at certain life stages) in influencing heart diseases from a life course perspective. Finally, the respondents were asked whether they had ever been diagnosed with heart-related conditions without accurate information of the onset age. Future longitudinal studies should be conducted to examine the role of childhood conditions in influencing both the onset and development trajectories of heart diseases in later life.

## Conclusion

This study investigated the influence of individuals’ childhood SES, health status, and health care resources during childhood on heart disease morbidity in later life. The findings suggested that all childhood varaibles except health care resources affected the morbidity of heart disease in later life directly in China, whereas health care sources during childhood might affect heart disease risk in later life indirectly through influencing adulthood health status. Therefore, identifying populations at risk of heart disease by using childhood conditions as screening criteria should be considered. Prevention strategies should also be implemented from a life course perspective.

## Data Availability

The datasets analysed during the current study are available in the CHARLS repository, [http://charls.pku.edu.cn].

## Abbreviations

BMI: Body mass index
CHARLS: China Health and Retirement Longitudinal Study
MICE: Multiple imputation by chained eqs.
OR: Odds ratio
SRH: Self-rated health
SES: Socioeconomic status
